# Illinois State Medical Society

**Published:** 1885-06

**Authors:** 


### Editorial.

### Thirty-Fifth Annual Meeting of the Illinois State Medical Society.

The thirty-fifth annual meeting of the Illinois State Medical Society, held at Springfield, on the 19th, 20th, and 21st of May, was marked by many pleasant features. The work of the general and special committees was well performed, although there was a notable absence of several chairmen.

Few papers, containing the results of original investigation, were read, and for the prizes established last year, at the meeting of the Society in Chicago, no papers were offered to the committee.

The Society has ample funds to encourage original research, and it is to be regretted that a portion of the surplus of nine hundred dollars, reported by the Treasurer as remaining in the treasury, should not have been expended in this manner.

The membership of the Society numbers over four hundred. About one hundred and forty delegates and permanent members responded to the roll-call. We sincerely hope that the representation, next year, at Bloomington, will be more nearly adequate, from a numerical point of view.

The reports of the Committees on Medical Legislation and State Medicine were encouraging in the highest degree. This fact is due to the arduous labors of the members of the committees and to the unusually large representation of the profession in the Senate and House. Four members of the regular profession are State Senators, and eight, members of the House.

It is believed that both the Anatomy Bill and the "Campbell" Bill, relative to the commitment of the insane, will become laws before the present session closes.

While discussions were neither frequent nor brilliant, there was a pleasurable absence of all bickering and wrangling.

The social character of the meeting was maintained to an eminent degree. His Excellency, General Richard J. Oglesby Governor of the State of Illinois, and Mrs. R. J. Oglesby, tendered the members of the Society a reception on Tuesday evening. On Wednesday evening, receptions were held at the residences of Dr. and Mrs. B. M. Griffith, Dr. and Mrs. F. L. Matthews, Dr. and Mrs. H. B. Buck.

